# Highly Potent Immunotoxins Targeting the Membrane-distal N-lobe of GPC3 for Immunotherapy of Hepatocellular Carcinoma

**DOI:** 10.7150/jca.66978

**Published:** 2022-02-14

**Authors:** Jingwen Li, Lanxin Xiang, Qian Wang, Xuqian Ma, Xin Chen, Yuankui Zhu, Yaxi Yang, Le Huang, Huixia He, Lilei Xu, Xinjun Liang, Shuang Dong, Sheng Hu, Hanjie Li, Mingqian Feng

**Affiliations:** 1 College of Life Science and Technology, Huazhong Agricultural University, Wuhan, Hubei 430070, China.; 2 Beijing Advanced Innovation Center for Structural Biology, School of Life Sciences, Tsinghua University, No. 30 Shuangqing Road, Beijing 100084, China.; 3 Department of Internal Medicine-Oncology, Hubei Cancer Hospital, Wuhan, Hubei 430070, China.; 4 CAS Key Laboratory of Quantitative Engineering Biology, Shenzhen Institute of Synthetic Biology, Shenzhen Institutes of Advanced Technology, Chinese Academy of Sciences, Shenzhen, Guangdong 518055, China.; 5 College of Biomedicine and Health, Huazhong Agricultural University, Wuhan, Hubei 430070, China.

**Keywords:** GPC3, immunotoxin, monoclonal antibody, epitope mapping, hepatocellular carcinoma

## Abstract

Glypican-3 (GPC3) has become a compelling target for immunotherapy of hepatocellular carcinoma, including antibody-drug conjugate (ADC), and ADC-like immunotoxin. To investigate the impact of epitopes on the potency of ADCs, current study generated a large panel of chicken monoclonal antibodies (mAbs) that targeted 12 different and over-lapping epitopes on GPC3. These mAbs demonstrated a very high affinity with K_d_ values in the range of 10^-9^-10^-14^ M, and the highest affinity (K_d_ value of 0.0214 pM) was 40-fold higher than the previously generated high-affinity mAb YP7 (K_d_ value of 0.876 nM). Additionally, these mAbs exhibited excellent thermostability with T_m_ values in the range of 45-82 °C. As a proof-of-concept study for ADC, we made immunotoxins (scFv fused with PE24, the 24-kDa cytotoxic domain of *Pseudomonas* exotoxin A) based on these mAbs, and we found that immunotoxins targeting the N-lobe of GPC3 were overall much more potent than those targeting the C-lobe and other locations. One representative N-lobe-targeting immunotoxin J80A-PE24 demonstrated 3 to 13-fold more potency than the hitherto best immunotoxin HN3-PE24 that was previously developed. J80A-PE24 could suppress tumor growth much greater than HN3-PE24 in a xenograft mouse model. Combination of J80A-PE24 with an angiogenesis inhibitor FGF401 showed additive effect, which dramatically shrank tumor growth. Our work demonstrated that, due to high affinity, excellent thermostability and potency, chicken mAbs targeting the N-lobe of GPC3 are appealing candidates to develop potent ADCs for immunotherapy of liver cancer.

## Introduction

Liver cancer ranks as the sixth most common cancer and the third most lethal malignancy 1. Hepatocellular carcinoma (HCC) is the major form of primary liver cancer. The most effective therapies for HCC are resection, liver transplantation, and local ablation, which, however, are only suitable for 15% to 20% of HCC cases that are diagnosed at an early stage 2, and the 5-year recurrence rate is as high as around 70% 3. Sorafenib was the first targeted drug approved in 2007 that can be used in first-line settings for patients diagnosed with advanced HCC or patients who progressed into this stage after the failure of other therapies 4. But sorafenib could only increase the median survival time by approximately 3 months due to the drug resistance that eventually develops 5, 6. It would be as long as a decade before another targeted drug, Lenvatinib, that showed non-inferiority in comparison with sorafenib, was approved for HCC treatment 4. Thereafter, regorafenib, cabozantinib, ramucirumab, and two programmed cell death protein 1 (PD-1) inhibitors, pembrolizumab and nivolumab which demonstrated objective response rates of 15-20%, were approved in the second- and third-line settings for advanced HCC 4, 7. Tremelimumab, a mAb against cytotoxic T-lymphocyte-associated protein 4 (CTL-4), demonstrated a partial response rate of 17.6% and disease control rate of 76.4% in HCC patients in a recent clinical trial 8. Despite the progress of these targeted therapies and immunotherapies, the 5-year survival rate of HCC still remains as low as 18% 2, and an urgent need for more effective therapies are desperately required for HCC patients.

GPC3 is an appealing target for HCC immunotherapy 9. Currently only three classes of GPC3 mAbs with determined epitopes have been described. Mouse-derived mAbs GC33 and YP7 represent one class that targets the cell surface-proximal C-terminal region of GPC3 (a.a. 510-560) 10, 11. HN3, a fully human heavy chain variable region (VH) domain antibody, targets the N-lobe of GPC3 and is able to block Wnt signaling to inhibit HCC cell proliferation 12, 13. A recent report identified another human mAb, 32A9, that recognizes the middle lobe of GPC3 (around the last predicted helix, spanning a.a. D464-R474) 14.

Several GPC3-targeting immunotherapies based on afore-mentioned mAbs have demonstrated encouraging results in preclinical or early clinical studies, including bispecific antibody, chimeric antigen receptor T cell (CAR-T), and ADC. Bispecific antibody ERY974, constructed from GC33, was highly effective in killing various types of tumors 15, and phase 1 clinical trial is in progress (NCT02748837). Phase 1 clinical trial of GPC3 CAR-T therapy based on GC33 showed that the overall survival rates at 3 years, 1 year, and 6 months were 10.5%, 42.0%, and 50.3%, demonstrating a modest antitumor activity of CAR-T cells in patients with advanced HCC 2. Dan Li et al. revealed that the CAR-T based on YP7 eliminated tumors in 66% of mice by week 3, whereas CAR-T based on HN3 did not reduce tumor burden 16. The first GPC3-targeting ADCs, hYP7-PC and hYP7-DC, were developed with potency at picomolar concentrations against a panel of GPC3 positive cancer cells 17. Although these immunotherapies are promising for HCC, there are still various challenges before they eventually benefit patient 18-20.

Immunotoxins provide another option in cancer treatment and have achieved FDA approval in several cases 21. The CD22-targeting immunotoxin Lumoxiti was recently approved with an objective response rate of 86% and a complete response rate of 57% in a phase 1 study of patients with relapsed/refractory HCL 22. Afore-mentioned GPC3 mAbs were used to generate a series of recombinant immunotoxins to test their potential clinical applications for HCC. The HN3-PE38 (the 38-kDa cytotoxic domain of *Pseudomonas* exotoxin A) showed stronger antitumor activity, with IC_50_ values around 3 ng/ml, than YP7-PE38 (IC_50_ values around 10 ng/ml) on GPC3-overexpressing G1 cells, although the affinity of HN3 is much lower than that of YP7, suggesting that the epitopes recognized by HN3 and YP7 dominate the difference of their activity 23-25. Another immunotoxin 32A9-PE24 exhibited much weaker cytotoxicity, with IC_50_ values around 40 ng/ml on GPC3-over-expressing G1 cells 14. Based on the available data, HN3 immunotoxin was most potent, which is also consistent with the data of the current study. Apart from HN3 immunotoxin, it would be worthwhile to explore more potent immunotoxins by targeting other epitopes of GPC3.

GPC3 is a highly conserved orthologue protein in mammals. It is challenging to generate GPC3 mAbs against the conserved epitopes by immunization of mammals (e.g. rodents and rabbit). The phylogenetical distance between mammals and birds offers more possibilities to produce chicken mAbs with higher affinity and broad epitope coverage against conserved mammal homolog proteins **26**. Here, we described the isolation and characterization of a large panel of GPC3 mAbs by immunization of chicken and phage display. These mAbs covered 12 epitopes, with majority of them recognizing epitopes on the N-lobe and C-lobe of GPC3. Based on these mAbs, we made immunotoxins (scFv-PE24 format), and found that most of the N-lobe targeting immunotoxins were much more potent than those targeting the C-lobe and other locations. *In vivo* studies demonstrated that the most potent one of the N-lobe-targeting immunotoxins, J80A-PE24, could transiently control Hep3B tumor growth in NSG (NOD, Prkdc^scid^, IL2rg null) mice, which was further significantly boosted through combination with FGF401, a clinically testing fibroblast growth factor receptor 4 (FGFR4) inhibitor that has activity of multiple tyrosine kinase-inhibition and anti-angiogenesis, leading to a much longer survival time of the treated mice. Collectively, our study encourages the development of N-lobe targeting immunotoxins, especially J80A, for treatment of HCC.

## Materials and Methods

### Cell lines

GPC3 positive HCC cell lines HepG2, Hep3B, and Huh-7, and GPC3 negative A431 cell line were used in the current study. An artificial G1 cell line was created by lentiviral transduction of A431 cells to stably over-express full-length GPC3. All cell lines were maintained as adherent monolayer cultures in DMEM medium (Invitrogen, Carlsbad, CA) supplemented with 10% fetal bovine serum (HyClone, Logan, UT), 1% L-glutamine (Invitrogen, Carlsbad, CA), and 1% penicillin-streptomycin (Invitrogen, Carlsbad, CA), and incubated in 5% CO2 with a balance of air at 37 °C. Cells were passaged twice a week with refreshed medium. For cytotoxicity assays, aforementioned cell lines were stably transduced to constitutively express firefly luciferase (ffLuc2)-EGFP fusion gene, and the resulting cell lines were renamed HepG2LG, Hep3BLG, Huh-7LG, A431LG, and G1LG. Cell survival was indicated by the intracellular luciferase activity.

### Preparation of recombinant GPC3, mAbs, and immunotoxins

Recombinant human and mouse GPC3 were derived from NP_004475.1 and NP_057906.2, respectively. The extracellular domain (a.a. 25-550) of both species was fused with 6 × His tag (GPC3-His) for purification purpose and expressed in HEK 293F cells. A shortened form of human GPC3 (a.a. 25-480) was fused with human IgG1 Fc (GPC3-hFc) and prepared for the immunization and the library screening. Point mutants of human GPC3 (a.a. 25-480) were also created as hFc fusion protein as indicated in the fine epitope mapping experiment. The native secretion signal peptide of GPC3 (a.a. 1-24) was replaced with IL-2 secretion signal for enhanced secretive expression and purification. A 64-aa fragment of mesothelin (NP_005814.2, a.a. 296-359), named as IAB domain in a previous study 27, was fused with hFc and used as an hFc tag control. Aforementioned expression cassette was cloned into mammalian expression vector pFUSE, expressed in 293F cells (Invitrogen) in a secretive manner, and purified via nickel or protein A affinity chromatography. GPC3 mAbs of the current study were expressed as scFv-hFc format in the same way. Immunotoxins were expressed as His-scFv-PE24 format in E.coli BL21 (λDE3) following the published protocol with some modifications 24. Briefly, the engineered PE24 fragment was fused at the C-terminal of the scFv, and 6 × His was fused at the N-terminal of scFv for purification purpose. The whole expression cassette was cloned into the pET28a vector (Novagen, Madison, WI, USA) with EcoRV and HindIII restriction enzymes. The Escherichia coli BL21 (λDE3) strain was transformed with the expression plasmid, cultured in the medium containing kanamycin, shaking at 37 °C at the speed of 220 rpm. When OD600 reached 0.6, a final concentration of 0.5 mM IPTG was added to the culture, followed by continued shaking for 8 h at 220 rpm and 30 °C. After centrifugation to harvest the cells, the pellet was resuspended in PBS buffer, disrupted by a high-pressure homogenizer. After clarifying the cell lysate by centrifugation, immunotoxin from the supernatant was purified via nickel column chromatography.

### Phage display and panning of GPC3 binders

The library had an estimated size of 2 × 10^9^ original clones. Purified GPC3-hFc was immobilized on 96-well plate for the panning process following the previous protocol 12. To exclude hFc binders during the panning process, IAB-hFc was always included in the blocking buffer at a final concentration of 100 μg/ml. After four rounds of panning, a total of 8 plates (768 clones) were randomly picked. To identify GPC3 specific binders, recombinant GPC3-hFc or IAB-hFc control was immobilized on 96-well plate at 5 μg/ml. After blocking with PBST buffer containing 0.5% BSA, the coated plate was incubated with 50 μl pre-blocked phage solution at 37 °C for 30 min. Phage binding was detected with HRP-conjugated anti-M13 antibody (11973-MM05T-H, Sinobiological, China). GPC3 specific binders will show at least 5-fold higher binding signal to GPC3-hFc than IAB-hFc control. A total of 700 GPC3 specific binders were identified and sequenced, and 245 different scFv sequences were finally obtained.

### Epitope binning and mapping

For the epitope binning analysis, competitive phage ELISA was carried out. The most highly enriched clone A5 was chosen as the first reference binder for the ELISA. Recombinant A5 scFv-hFc was prepared and immobilized on 96-well plate at 5 μg/ml. After washing and blocking, the plate was incubated with 1 μg/ml GPC3-His. After washing again, 50 μl phage binders were added to the plate. Any phage binders that have different epitopes from A5 will show binding signal after the subsequent color development with HRP-conjugated anti-M13. As a parallel positive control, GPC3-His was directly immobilized on the plate and incubated with same amount of phage binders. A suppression ratio was calculated by the percentage of the decrease in the competitive binding compared with the direct binding. Any phage binders with suppression ratio less than 50% were regarded as new reference binders that had epitopes different or diversified from the coating mAb. The whole process was repeated over and again, and finally a total of 14 epitopes were narrowed down.

To further confirm the 14 epitopes at protein level, competitive protein ELISA was performed based on the recombinant mAbs. All of the 14 reference mAbs were immobilized on the ELISA plate at 5 μg/ml, and GPC3-His was added to the plate at 1 μg/ml. Subsequently, each of the reference mAbs was biotinylated and separately incubated with the plate at increasing concentrations. Binding of the biotinylated mAbs to the captured GPC3 was detected with streptavidin-HRP conjugate (D111054, Sangon Biotech, China). Any mAb that failed the binding indicated it shared the epitope with the corresponding coating mAb. As a positive control, GPC3-His was also directly coated on the plate at 1 μg/ml and detected by the biotinylated mAb.

To determine the precise binding locations, a series of GPC3 point mutants were created, which covered the potentially contacting residues that were predicted by structural analysis of the GPC3-mAb complex. Binding intensity of the mAbs to the wild type and mutant GPC3 was measured by ELISA as follows. A 96-well plate was coated with 12 recombinant immunotoxins at 5 μg/ml, and then the wild type and mutant GPC3-hFc were added to the plate at 1 μg/ml. Binding of GPC3-hFc was detected with HRP-conjugated goat anti-human Fc (SSA001, Sinobiological, China).

To plot the protein binding curves of the mAbs, purified human and mouse GPC3-His was captured on the 96-well plate at 5 μg/ml in PBS buffer, 50 μl per well, at 37 °C for 30 min. After the plate being blocked, increasing amount of mAbs (scFv-hFc format) was added to the plate and incubate at 37 °C for 30 min. After washing twice with PBST, binding of the mAbs was detected by HRP-conjugated goat anti-human Fc. The apparent binding affinity (EC_50_ value) was determined by Prism software.

To plot the protein binding curves of the immunotoxins, purified GPC3-hFc was coated on the 96-well plate at 5 μg/ml at 37 °C for 30 min. After washing and blocking with PBST buffer containing 0.5% BSA, the plate was incubated with increasing concentrations of immunotoxins (His-scFv-PE24 format), and immunotoxin binding was detected by HRP-conjugated mouse anti-His antibody (105327-MM02T-H, Sinobiological, China).

### Structural analysis

The structures of human GPC3 and the mAbs (scFv-His) were modeled by using the online tool SWISS-MODEL (https://swissmodel.expasy.org/) with the default settings.

### Affinity measurement by SPR

To determine the binding kinetics of the mAbs, SPR was conducted on Biacore T200 instrument at 25 °C. Recombinant GPC3-his was coupled to a CM5 sensor chip using the standard amine coupling procedure at a target density of 100 response units (RU). PBST buffer (PBS buffer containing 0.05% tween 20, pH 7.4) was utilized as a running buffer. The 2-fold gradient diluted scFv-hFc in running buffer was injected over the flow cell for 180 s at a flow rate of 30 µl/min. The dissociation time was 600 s. Kinetic rate constants were obtained by curve fitting according to a 1:1 binding model using Biacore T200 Evaluation.

### Measurement of thermostability

GPC3 mAbs with a concentration of 50 μg/ml in PBS were assigned into glass capillaries and placed in the sample chamber of a Prometheus NT.48 differential scanning fluorometer (NanoTemper Technologies, Inc, Munich, Germany). Samples experienced a time-dependent temperature gradient over 20-95 °C at a rate of 1.5 °C /min. Fluorescence emission at 330 nm and 350 nm (excitation wavelength, 295 nm) were recorded. Their first derivatives are presented as a function of the applied linear thermal ramp. The T_m_ value is defined as the temperature at the maximum first derivative of the ratio of fluorescence emission at 350 and 330 nm (F350/F330). The T_m_ value was determined by a polynomial that fits the temperature-fluorescence ratio curve implemented in the manufacturer's software.

### Flow cytometry

Cells were detached with trypsin-EDTA (ThermoFisher, Waltham, MA), washed, and resuspended in cold PBS buffer containing 5% BSA. One million cells were co-incubated with GPC3 mAbs (1 μg/ml, or as indicated in specific experiments). The antibody binding was detected by Allophycocyanin (APC)-conjugated goat anti-human IgG (Jackson ImmunoResearch Inc, West Grove, PA). The fluorescence intensity (Geo. Mean) was measured by using BD FACS Aria. To confirm the cell-binding activity of immunotoxins, one million G1 cells were incubated with 10 μg/ml immunotoxin on ice for 1 hour. The binding of immunotoxin was detected by APC-conjugated mouse anti-His antibody (BioLegend).

### *In vitro* cytotoxicity assay

Cytotoxicity of the immunotoxins was measured by the firefly luciferase reporter assay. Cancer cells were stably transduced with a lentiviral vector that carries a luciferase reporter gene. Ten thousand cells were seeded in a 96-well cell culture plate (200 μl per well), supplemented with purified immunotoxins at the indicated final concentrations, and incubated at 37 °C for 3 days. Untreated cells were used as control. Cell viability was determined by quantifying the enzymatic activity of the intracellular luciferase that was released by two rounds of freezing-thawing.

### *In vivo* study

All mice were housed and treated under the protocol approved by Animal Care and Use Committee of Huazhong Agricultural University. Six to eight weeks NSG mice were subcutaneously inoculated with five million Hep3B cells. When the average tumor size reached 200-250 mm^3^, mice were treated with immunotoxins via the tail vein injection every other day. PBS solvent was used as control. Tumor dimensions were determined every two or three days by using a caliper. Tumor volume (mm^3^) was calculated by the formula V = ab²/2, where a and b are tumor length and width in millimeters, respectively. In some groups, mice were treated with FGFR4 inhibitor FGF401 (MCE, Shanghai, China) by oral delivery at dose of 30 mg/kg and given twice a day. FGF401 was prepared by suspending in 0.5% CMC-Na at 6 mg/ml.

### Statistical analysis

All statistical analyses were conducted using GraphPad Prism 5 (GraphPad Software, Inc., La Jolla, CA). Differences between two groups were analyzed using the unpaired Student's t-test of means (two-tailed), with P value < 0.05 defined as statistically significant. Comparisons among three or more groups were performed using one-way ANOVA.

## Results

### Generation and epitope mapping of the GPC3 chicken mAbs

**Figure 1:**
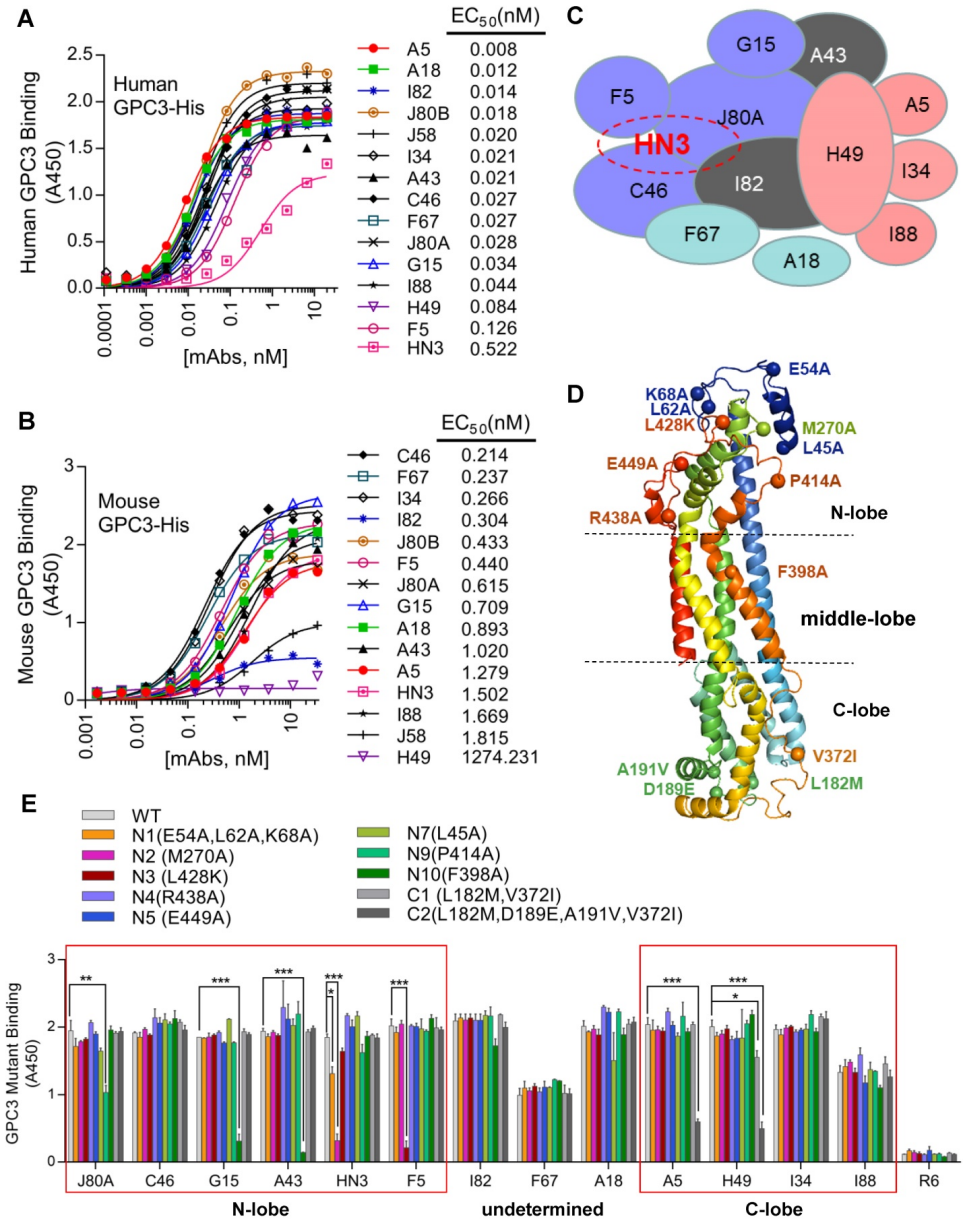
** Generation and fine epitope mapping of GPC3 mAbs.** (**A-B**) Binding of the mAbs to human (**A**) and mouse GPC3 (**B**). ELISA plate was coated with human or mouse GPC3-His (5 µg/ml in PBS, 50 µl/well), followed by incubation with increasing amount of the mAbs (scFv-hFc format). Antibody binding was detected by HRP-conjugated goat-anti-human Fc. EC_50_ values were calculated by Software GraphPad Prism using non-linear regression (hyperbola two-site binding). (**C**) Schematic diagram illustrating the inter-relationships of the 12 epitopes that have been identified, which formed two epitope clusters, J80A and H49. Purple indicated J80A cluster (J80A, G15, F5, and C46); pink indicated H49 cluster (H49, A5, I34, and I88); brown indicated epitopes cross with J80A and H49 cluster (A43 and I82); blue indicated epitopes cross neither J80A nor H49 cluster (F67 and A18). The shapes, sizes and their overlapping areas of the ellipses shown in the figure were not of special significance. (**D**) Location of the residues of human GPC3 that were mutated to confirm the binding site of the mAbs. Five of these residues, L182M, D189E, A191V, N210S, V372I (first letter indicated human and followed by mouse), were diverged from human to mouse. The structure was modeled by web tool SWISS-MODEL (https://swissmodel.expasy.org/) using the default parameters. (**E**) Binding of the mAbs to GPC3 mutants. Recombinant immunotoxins made from the mAbs were immobilized on the ELISA plate at 5 µg/ml. Wild type (WT) and mutant GPC3-hFc were incubated with the plate at 1 µg/ml. Binding of GPC3 was detected with HRP-conjugated goat-anti-human Fc. R6, a non-targeting immunotoxin to EGFRvIII generated in our lab by immunizing rabbits with an EGFRvIII peptide, was served as isotype control. *P<0.05, **P<0.01, ***P<0.001, one-way ANOVA.

A phage-displayed scFv library was constructed from the splenic mRNA of the chickens immunized with GPC3-hFc as previously described 28. After 4 rounds of panning, 700 GPC3-specific binders were selected, which were initially binned to 14 the epitopes by competitive phage ELISA following the previous protocol 29. These 14 chicken mAbs bound GPC3 much stronger than HN3 (Figure 1A), a previously developed human VH domain antibody to GPC3 12. Majority of the mAbs had calculated EC_50_ values in the range of 0.01-0.08 nM, while HN3 had an EC_50_ of 0.5 nM. All mAbs, except for H49, could bind mouse GPC3 (Figure 1B), with the calculated EC_50_ values in the range of 0.2-1.8 nM. Further competitive protein ELISA narrowed down the epitopes of the 14 mAbs to 12 unique and over-lapping epitopes (Figure 1C), which formed two epitope clusters, with J80A and H49 being located in the center of each cluster. Four mAbs (J80A, F5, C46, and I82) had over-lapping epitopes as HN3, a previously developed VH-only mAb that could directly inhibit HCC cell proliferation.

To determine the spatial locations of the epitopes, a series of GPC3 point mutants were created based on structural analysis. Considering H49 did not bind mouse GPC3, and that five out of six mouse GPC3 residues (L182M, D189E, A191V, N210S, V372I, first letter indicated human and followed by mouse) were located in the very end of the C-lobe, it was reasonable to postulate that H49 and H49-competing mAbs (A5, I34, I88) may bind to the C-lobe of GPC3. In contrast, previous study had already determined that HN3 bound to the very end of N-lobe of GPC3 13, therefore it was likely that HN3-competing mAbs (J80A, F5, C46, and I82) may also bind to the N-lobe. Based on these postulation, key residues that were potentially required for the mAbs binding were mutated to alanine or charged lysine (L428K) (Figure 1D). Binding analysis of the mAbs to the wild type and mutant GPC3 identified the key residues that were required for the binding of the mAbs (Figure 1E). Key residues that were essential for H49 and A5 binding were determined by C-lobe quadruple mutant (L182, D189, A191, V372), while N-lobe mutant N2 (M270A), N9 (P414A), N3(L428K), and N10 (F398A) almost totally abolished HN3, J80A, F5, G15 and A43 binding. Since C46 competed with both HN3 and J80A, it presumably binds somewhere in the N-lobe. Similarly, I34 and I88 presumably bind somewhere in the C-lobe since both of them only competed with H49. Binding sites of other mAbs (I82, F67, and A18) have not yet been determined.

### Binding properties and thermal stability of the GPC3 mAbs

Protein binding kinetics of the mAbs was measured by surface plasmon resonance (SPR). The results revealed that all mAbs but F67 had sub-nanomolar to sub-picomolar affinities for GPC3 (Table 1). A18 exhibited the highest affinity, with a K_d_ value of 0.0214 pM that was 40-fold higher than the previously generated high-affinity mAb YP7 (K_d_ value of 0.876 nM) 30. HN3 demonstrated the lowest affinity with a K_d_ value of 1.95 nM, which was very close to F67 (K_d_ value of 1.45 nM).

**Figure 2:**
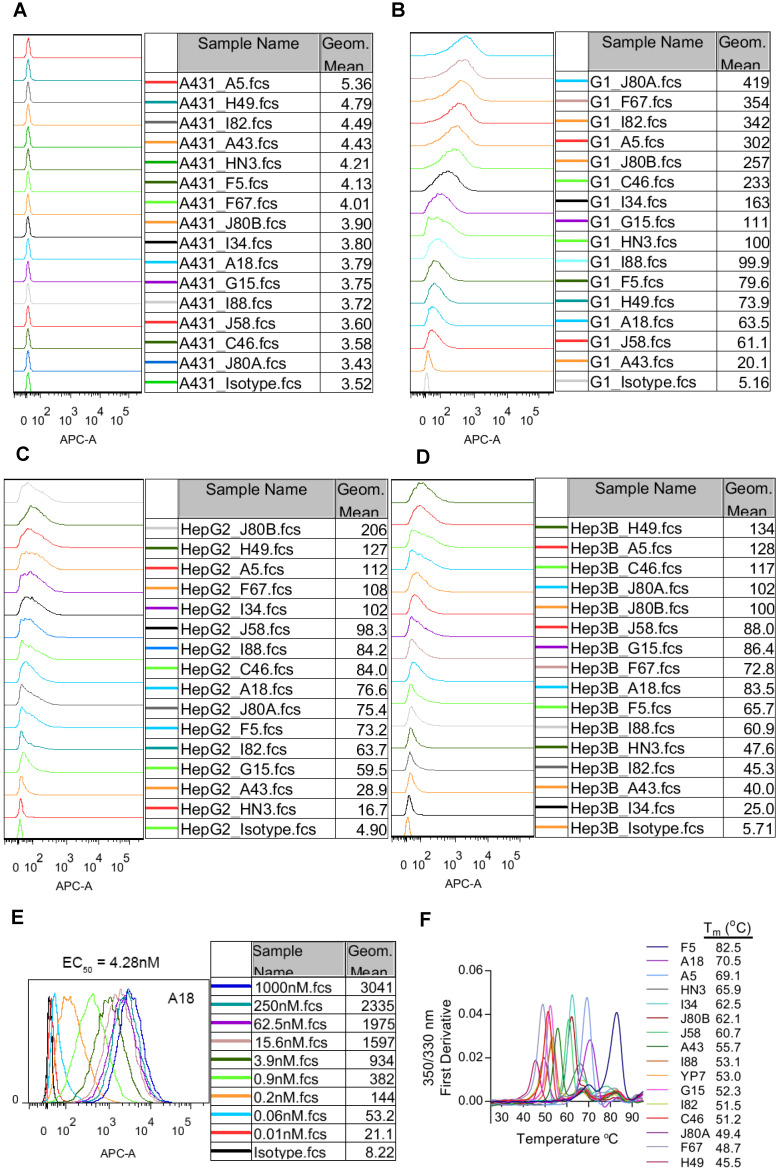
** Binding properties and thermostability of the GPC3 mAbs.** (**A**) Binding of the mAbs to GPC3 negative A431 cells. Cells were incubated with 1 µg/ml of the mAbs on ice for 1 hour. Antibody binding was detected by APC-conjugated goat-anti-human IgG. Pooled human IgG was used as isotype control. (**B**) Binding of the mAbs to GPC3 over-expressing A431 cells (named G1). Experimental conditions were same as **A** except that 0.01 µg/ml mAbs was used. (**C-D**) Binding of the mAbs to GPC3 positive HCC cell lines HepG2 (**C**) and Hep3B (**D**). Experimental conditions were same as **B**. (**E**) Cell binding EC_50_ values of exemplary mAbs A18. EC_50_ values were measured on G1 cells and calculated by using software GraphPad Prism. (**F**) Thermostability of the mAbs. T_m_ values were measured by nanoDSF (Prometheus NT.48) instrument.

Cell binding affinity and specificity of the mAbs was evaluated by flow cytometry on three GPC3 positive HCC cell lines (HepG2 and Hep3B), GPC3 negative A431 cells, and GPC3-overexpressing A431 cells (named G1). All mAbs exhibited specific binding to GPC3 positive cells and no background binding to GPC3 negative A431 cells (Figure 2A-D). We also measured the cell binding affinity of A18, which had protein binding K_d_ values of 0.0214 pM (Table 1), and the calculated EC_50_ values of cell binding were 4.28 nM (Figure 2E).

The thermostability of the mAbs was represented by the transition temperature for thermal denaturation, which was measured by differential scanning fluorimetry. The melting curve of the mAbs was shown in Figure 2F. More than 40% of the mAbs demonstrated excellent thermostability with T_m_ values exceeding 60 °C, and the most stable mAb F5 had T_m_ value reaching to 82.5 °C.

### Inhibition of HCC cell proliferation by mAb H49

**Figure 3:**
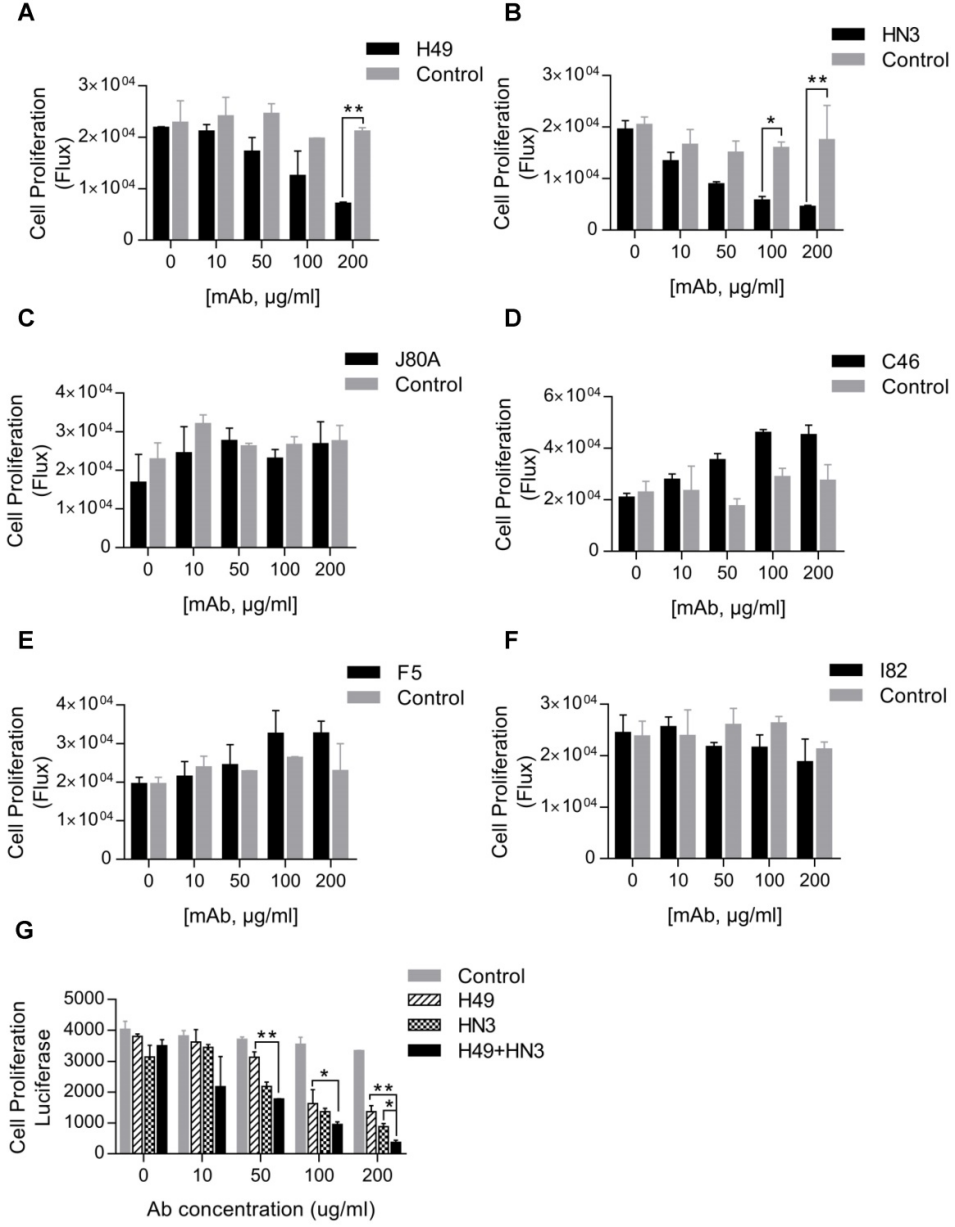
** Suppression of HCC cell proliferation by the GPC3 mAbs.** One thousand Hep3BLG cells were seeded on 96-well plate, and incubated with the corresponding mAbs at the indicated final concentrations for 5 days. Cell proliferation was quantified by measuring the intracellular luciferase activity of Hep3BLG cells. Untreated cells were used as control. *P<0.05, **P<0.01, unpaired Student t-test.

Our initial interest was to explore GPC3 mAbs that had stronger potency than HN3 in suppressing HCC proliferation. Surprisingly, only H49 that recognized the C-lobe of GPC3 moderately inhibited the growth of Hep3B, but was less active than HN3 that targeted the N-lobe of GPC3 (Figure 3A-B). Interestingly, although mAbs J80A, C46, F5, and I82 had epitopes overlapping with HN3, none of them could inhibit HCC cell proliferation (Figure 3C-F). However, the combination of H49 and HN3 demonstrated an additive effect in suppression of HCC proliferation (Figure 3G).

### Construction of GPC3 immunotoxins

**Figure 4:**
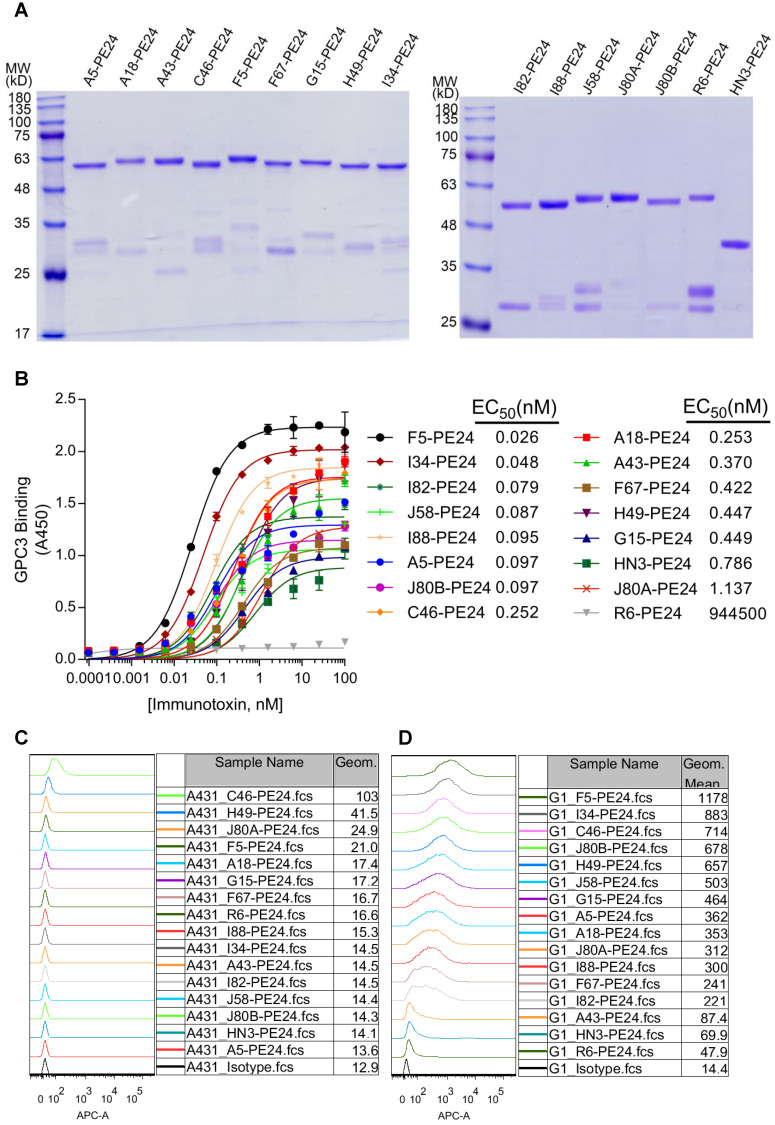
** Construction of GPC3 immunotoxins.** (**A**) SDS-PAGE analysis of the recombinant immunotoxins. Two micrograms of protein were loaded for each lane. (**B**) Measurement of immunotoxins binding to GPC3. ELISA plate was coated with 5 µg/ml GPC3-hFc, and incubated with increasing amounts of immunotoxins. Binding of immunotoxin was detected by HRP-conjugated moue anti-His antibody. EC_50_ values were calculated by using software GraphPad Prism. R6-PE24 was used as non-targeting immunotoxin control that specifically bound to EGFRvIII. (**C-D**) Flow cytometry analysis of the immunotoxins binding to GPC3 negative A431 (**C**) and GPC3 over-expressing G1 cells (**D**). One million cells were incubated with 10 µg/ml immunotoxin. Binding of immunotoxin was detected by APC-conjugated mouse-anti-His antibody. R6-PE24 was used as an isotype control.

Given the moderate activity of tumor growth inhibition, but high affinity and broad epitope coverage of the mAbs, it would be interesting to investigate the potency of these mAbs-derived ADCs that generally require high affinity. For a proof of concept, we made immunotoxins that fused the scFv with pseudomonas exotoxin PE24 and 6 × His tag (His-scFv-PE24). Previously developed HN3-PE24, the hitherto most potent immunotoxin to kill GPC3 positive cells 23, was included as comparison. All immunotoxins were expressed in E.coli and purified via affinity chromatography by the 6 × His tag. The purity was assessed by SDS-PAGE (Figure 4A).

Prior to the cytotoxicity assay, protein binding of the immunotoxins was confirmed by ELISA (Figure 4B), and the calculated EC_50_ values were ranging from 0.026 (F5-PE24) to 1.137 nM (J80A-PE24). Most of the immunotoxins (except for J80A-PE24) had higher protein binding affinity than HN3-PE24 (K_d_ value of 0.786 nM), and more than half of the immunotoxins had lower than sub-nanomolar affinity to GPC3.

Cell binding specificity of the immunotoxins was confirmed by flow cytometry on GPC3 negative A431 (Figure 4C) and GPC3-overexpressing G1 cells (Figure 4D). All immunotoxins had neglectable background binding on A431 cells, and much stronger binding on G1 cells. Most of the immunotoxins had stronger cell binding than HN3-PE24 (Figure 4D), except for A43 that had comparable cell binding as HN3.

### Cytotoxicity of GPC3 immunotoxins

**Figure 5:**
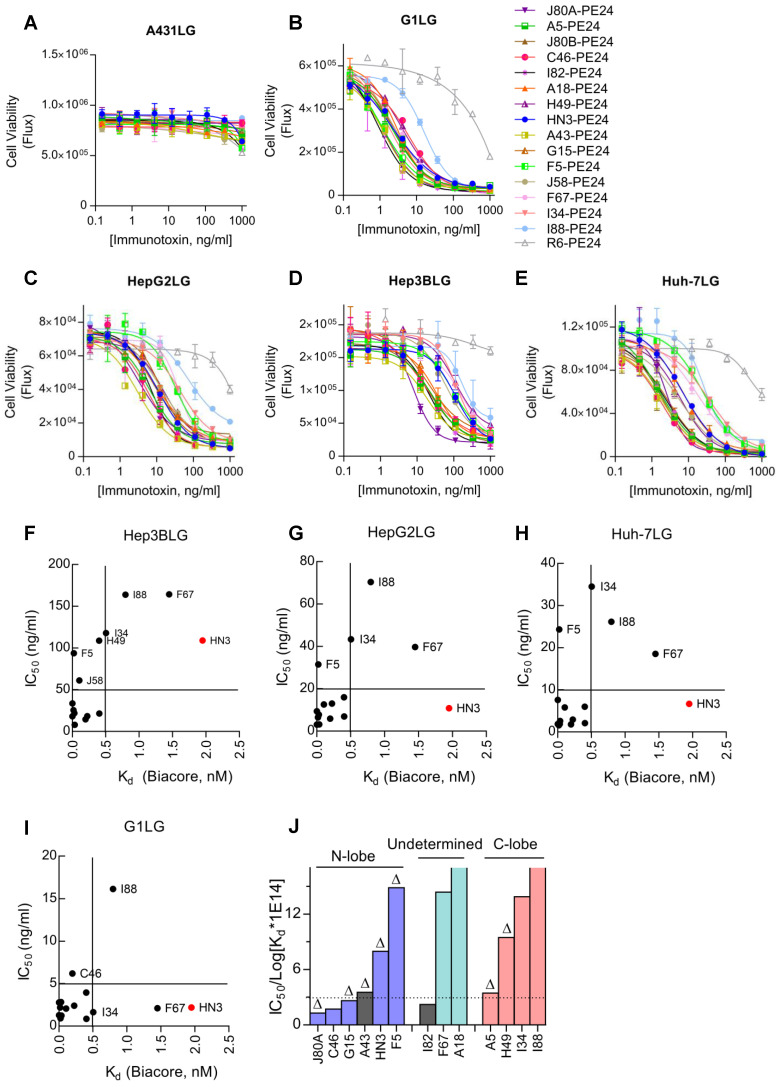
** Cytotoxicity of GPC3 immunotoxins. Cells were stably transduced to constitutively express a firefly luciferase gene (ffLuc2) as a reporter. Ten thousand cells were co-incubated with variable concentrations of immunotoxin for 72 hours. Cell viability was quantified by measuring the enzyme activity of the intracellular luciferase.** (**A**) Immunotoxin killing on GPC3 negative A431LG cells. R6-PE24 was an irrelevant immunotoxin to EGFRvIII. Data represent mean ± SD of triplicate. (**B**) Immunotoxin killing on G1LG cells, an engineered A431 that was constitutively over-expressing GPC3. (**C-E**) Immunotoxin killing on GPC3 positive Hep3BLG (**C**), HepG2LG (**D**), and Huh-7LG (**E**) cells. (**F-I**) Correlation analysis of IC_50_ values of the immunotoxins with the affinity of the corresponding mAbs (nM, measured by Biacore) on Hep3BLG (**F**), HepG2LG (**G**), Huh-7LG (**H**), and G1LG cells (**I**). (**J**) Averaged IC_50_ values of the immunotoxins normalized by transformed K_d_ values of the corresponding mAbs. Schematic illustration of the epitope map was attached for reference. The color in each bar corresponded to the Figure 1**C**. ∆ indicated key residues on GPC3 that were essential for binding of the corresponding mAb have been confirmed.

Cytotoxicity of the immunotoxins was measured on a panel of GPC3 positive cell lines by using a luciferase reporter assay. All immunotoxins had minimal killing on GPC3 negative A431LG cells (Figure 5A), and potent killing on GPC3-positive cells (G1LG, HepG2LG, Hep3BLG, and Huh-7LG) (Figure 5B-E). The calculated IC_50_ values of different immunotoxins on different cell lines were summarized and ranked based on Hep3BLG cells that had least sensitivity to immunotoxin killing (Table 2). Overall, the IC_50_ values of the immunotoxins were ranging between 1.0 to 100.0 ng/ml, depending on the tested cell lines. Surprisingly, 11 out of 14 immunotoxins were more potent than HN3-PE24 in Hep3BLG cells, and the most potent immunotoxin J80A-PE24 had an IC_50_ value of 7.9 ng/ml, which was 13-fold potent than HN3-PE24 (IC_50_ value of 109.0 ng/ml). For other cell lines (HepG2LG, Huh-7LG, and G1LG) that were more sensitive than Hep3BLG, majority of the immunotoxins (including HN3-PE24) showed comparable potency.

When an attempt to correlate the IC_50_ values with the absolute K_d_ values of the immunotoxins, there seemed no apparent correlations (Figure 5F-I). For majority of the immunotoxins, K_d_ values below 0.5 nM would generally bring down the IC_50_ values below 5-50 ng/ml depending on the cell lines (Figure 5F-I), suggesting that affinity below 0.5 nM could be a threshold for good potency of immunotoxins.

To deep understand the potential impact of epitope locations on the potency of immunotoxins, the averaged IC_50_ values of immunotoxins on three HCC cell lines (Hep3BLG, HepG2LG, and Huh-7LG) were normalized by their affinity (taking transformed K_d_ values) (Figure 5J). Herein, the transformed K_d_ values were obtained by multiplying the absolute K_d_ values (M) by 10^14^, followed by taking the logarithm values. As shown in Figure 5J, 4 out of 6 N-lobe-targeting immunotoxins (J80A, C46, G15, and A43) had the lowest normalized IC_50_ values below 3 ng/ml, while only 1 out of 4 C-lobe-targeting immunotoxins (A5) had normalized IC_50_ value around 3 ng/ml. The locations of I82, F67, and A18 had not been ascertained. The strong location bias suggested that N-lobe-targeting immunotoxins were much more potent than C-lobe-targeting immunotoxins.

### *In vivo* efficacy of GPC3 immunotoxins

**Figure 6:**
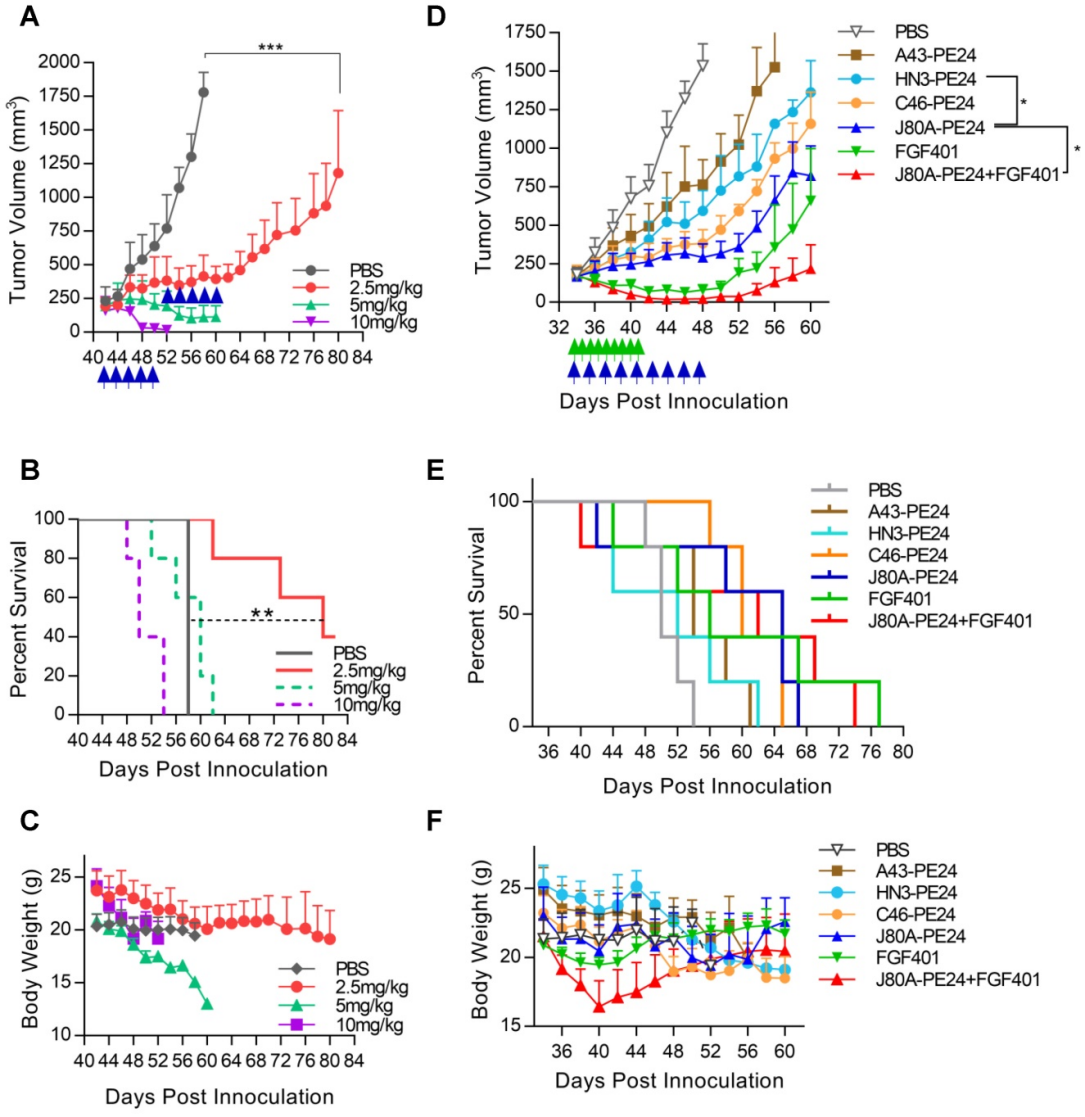
***In vivo* efficacy of GPC3 immunotoxins. NSG mice were subcutaneously inoculated with 5 × 10^6^ Hep3B cells. When tumor reached an average volume of 200-250 mm^3^, mice were treated with immunotoxins every other day, or with 30 mg/kg FGF401 given orally twice a day. Both tumor volume and body weight were measured every two or three days.** (**A**) Dose escalation experiment with immunotoxin J80A-PE24. Mice were treated with 2.5, 5, and 10 mg/kg immunotoxin by up to 10 intravenous injections. Arrows indicated injections. Values represent mean ± SEM (n = 5/group). ***P<0.001, unpaired Student's t-test. (**B**) Survival curves of mice treated in **A**. **P<0.01, Log-rank (Mantel-Cox) test. (**C**) Body weight of mice during treatment in **A**. (**D**) Comparison of different immunotoxins and chemotherapeutic inhibitor FGF401. Immunotoxin dose was fixed at 2.5 mg/kg. FGF401 suspension solution was orally delivered twice a day at 30 mg/kg and daily-based. Values represent mean ± SEM (n=5/group). *P<0.05, unpaired Student's t-test. (**E**) Survival curves of mice treated in **D**. (**F**) Body weight of mice during the treatment in **D**.

To evaluate the antitumor activity of immunotoxins *in vivo*, NSG mice were subcutaneously inoculated with Hep3B cells. After tumor formed and reached the volume of about 200-250 mm^3^, dose escalation experiment was performed with immunotoxin J80A-PE24. As shown in Figure 6A, treatment with dosage of 5 and 10 mg/kg led tumor regression; however, mice gradually died during the treatment due to intolerable toxicity. Treatment with 2.5 mg/kg was tolerated very well and could suppress tumor growth (Figure 6A), prolonging the survival time by about 24 days compared with PBS control (Figure 6B), and mice had a reversible body weight loss (Figure 6C). Therefore, 2.5 mg/kg was chosen as the maximal dose in the following-up experiments.

Based on the *in vitro* cytotoxicity potency, three top-ranking immunotoxins, J80A-PE24, C46-PE24, and A43-PE24, were chosen for the *in vivo* study to compare with each other and with HN3-PE24. We also evaluated the therapeutic effect of a reversible FGFR4 inhibitor, FGF401, in monotherapy and in combination with J80A-PE24. As shown in Figure 6D, J80A-PE24 was significantly more potent than HN3-PE24. Combination of FGF401 with J80A-PE24 significantly improved efficacy than J80A-PE24 monotherapy, leading to temporal tumor disappearance in some treated mice. However, tumor relapse still occurred after the treatment was stopped. In terms of survival time (Figure 6E), FGF401 and its combination with J80A-PE24 gave the longest survival time, followed by J80A-PE24.

All immunotoxins caused progressive body weight loss (Figure 6F), but J80A-PE24 caused relatively less body weight loss than others. FGF401 caused slight body weight loss at a very short period of time. However, combination of FGF401 and J80A-PE24 had a rapid and dramatic body weight loss, suggesting an amplified side effect by the combination.

## Discussion

In recent years, chicken mAbs are gaining increasing attention for therapeutic purposes 31, 32, and a variety of pathogen-specific chicken mAbs have proved to have excellent therapeutic effects, such as *Pseudomonas aeruginosa* 33, *Helicobacter pylori* 34, (SARS)‑CoV 35, and (SARS)‑CoV-2 36. Owing to the higher affinity and broader epitope coverage compared with mammalian mAbs, chicken mAbs may be promising therapeutics to human cancers. Torben Gjetting et al. generated an anti-PD-1 chicken mAb, Sym021, with a stronger affinity (30 pM) compared to nivolumab and pembrolizumab 37. Janet Sim et al. identified approximately 70 mAbs targeting signal-regulatory protein α (SIRPα) with diverse sequence families and epitopes, and with high affinity ranging from low nanomolar to picomolar 38. Moreover, chicken antibodies displayed excellent thermal stability, retaining their neutralizing activity at 25 °C for one year, however, both sheep and mouse antibodies lost their activity within two weeks under the same conditions 39. These studies encouraged us to choose chicken as the host to make novel GPC3 mAbs that could cover as many diversified epitopes as possible.

The mAbs from the current study had very high affinity and excellent thermostability. Except for F67 that had a K_d_ value of 1.45 nM, all other mAbs had K_d_ values ranging from 0.795 nM to 0.0214 pM, which was 10-10,000 times higher than HN3 (K_d_ value of 1.95 nM). YP7 is another previously developed GPC3 mAb with super high affinity (K_d_ value of 0.876 pM in the current study) 30. However, the affinity of A18 was still 40 times higher than YP7. Most of our mAbs had much higher cell binding capacity than HN3 (Figure 2A-D). It should be noticed that the cell binding intensity did not exactly follow the same order of protein binding affinity (Table 1**,** Figure 2A-D), possibly due to the difference of the epitope accessibility for soluble and cell-surface GPC3. Moreover, most of them could bind mouse GPC3 with high affinity (Figure 1B), which makes it feasible to directly apply these mAbs in immune-competent mouse studies without the need to create surrogate molecules. More than 40% of the mAbs demonstrated excellent thermostability with T_m_ values exceeding 60 °C, and the most stable mAb F5 had T_m_ value reaching to 82.5 °C, which is a very important attribute when considering the possible biotechnological applications.

HN3 is the only reported mAb that can directly inhibit HCC cell proliferation 12. Interestingly, although mAb J80A, C46, F5, and I82 had epitopes overlapping with HN3, none of them could inhibit HCC cell proliferation (Figure. 3), which implied a sophisticated functional epitope located on the N-lobe of GPC3. HN3 is a VH-only domain antibody, which has a much smaller size than conventional scFv and probably only small-sized domain antibody could fit the mysterious HN3 epitope. H49 recognized the C-lobe of GPC3 and inhibited cell proliferation (Figure 3), suggesting that GPC3 have another new functional epitope located on the C-lobe, and this epitope might be relatively easier to be blocked by conventional scFv fragment such as H49.

It was reported that the epitope location on target had an effect on the potency of ADC. The ADC m906PBD targeting CD56 bound to the membrane-distal N-terminal IgG-like domains was much more potent than m900PBD bound to membrane-proximal domains for neuroblastoma cells, although the two antibodies share similar affinities *in vitro* 40. The immunotoxin HN3-PE38 targeting GPC3 showed stronger antitumor activity than YP7-PE38 with the higher affinity, suggesting that the epitopes recognized by immunotoxins dominate the antitumor activity 23. In this study, detailed epitope mapping analysis and point mutation studies identified two epitope clusters, J80A cluster (HN3, J80A, G15, A43, and F5) that targets the N-lobe, and H49 cluster (H49 and A5) that targets the C-lobe (Figure 1E). The rest antibody most likely binds to the N-lobe or C-lobe according to the competitive protein binding ELISA (Figure 1C). Cytotoxicity assays revealed that the N-lobe-targeting immunotoxins were generally more potent than C-lobe (Table 2, Figure 5J). Four out of six N-lobe-targeting immunotoxins, J80A, C46, G15, and A43, were on the top of the potency ranking list, while only one out of four C-lobe-targeting immunotoxins (A5) had comparable potency with the top-rankers (Table 2, Figure 5J). Taken together, membrane-distal N-lobe was a sweet spot to develop potent GPC3 immunotoxins, and J80A-PE24 might be in the center of the sweet spot, since nearly all J80A-overlapping immunotoxins (C46, G15, I82, and A43, except F5 and HN3) were very potent and ranked on the top of the potency list (Table 2, Figure 5J). The most potent immunotoxin J80A-PE24 demonstrated 13.7-fold more potency in cytotoxicity than the previously developed HN3-PE24 immunotoxin on Hep3B or 6.5-fold for potency normalized by their affinity on three HCC cell lines (Hep3BLG, HepG2LG, and Huh-7LG), which indicated J80A-PE24 was a potential candidate for further preclinical and clinical studies. Collectively, our study encourages and enables the development of immunotoxins targeting membrane-distal N-lobe of GPC3.

Immunotherapy-based combination is a promising strategy to control malignant cancers. Previous study tested the combination of HN3 immunotoxin with irinotecan in suppressing HCC tumor growth 23. Here, we chose a different chemotherapy FGF401, which is a FGFR4 inhibitor by blocking the binding of ATP to the kinase domain of FGFR4 to prevent FGF19/FGFR4 aberrant activation that leads to eventually activation of Ras-Raf-ERK1/2 MAPK and PI3K-Akt pathways contributing to HCC progression 41. FGF401 has remarkable anti-tumor activity in mice bearing HCC tumor xenografts and patient-derived xenograft models that were positive for FGFR4 42. Phase I/II study suggested that FGF401 had a manageable safety profile and promising clinical activity 43. However, it seemed that combination of FGF401 with immunotoxin did not have synergistic but instead additive effect (Figure 6D). Some of the treated mice in the combination group achieved temporal tumor disappearance. However, tumor relapse in the treated mice remained challenging.

To conclude, current study generated a large panel of chicken mAbs to GPC3 with broad epitope coverage. Most of the mAbs demonstrated super high affinity to both human and mouse GPC3 and excellent thermostability, with K_d_ values in the range of 10^-9^-10^-14^ M and T_m_ values in the range of 45-82 °C. It was also found that the membrane-distal N-lobe of GPC3 was a sweet spot to develop potent immunotoxins, and one of such immunotoxins, J80A-PE24, was a compelling candidate to develop novel HCC therapeutics.

## Figures and Tables

**Table 1:** Affinity measurement of GPC3 mAbs by Biacore

Sample ID	K_on_ (1/Ms)	K_off_ (1/s)	Kd (M)
A18	9.73×10^6^	2.08×10^-7^	2.14×10^-14^
YP7	1.04×10^6^	9.15×10^-7^	8.76×10^-13^
A43	1.34×10^5^	2.04×10^-7^	1.52×10^-12^
A5	4.99×10^6^	9.05×10^-5^	1.82×10^-11^
F5	1.81×10^6^	4.13×10^-5^	2.28×10^-11^
J80B	2.16×10^6^	6.38×10^-5^	2.95×10^-11^
J80A	1.26×10^6^	4.41×10^-5^	3.50×10^-11^
J58	1.73×10^6^	1.82×10^-4^	1.05×10^-10^
C46	1.81×10^5^	3.54×10^-5^	1.96×10^-10^
G15	2.47×10^5^	5.57×10^-5^	2.25×10^-10^
H49	3.13×10^5^	1.25×10^-4^	4.00×10^-10^
I82	1.27×10^6^	5.12×10^-5^	4.05×10^-10^
I34	1.40×10^5^	7.07×10^-5^	5.04×10^-10^
I88	2.23×10^5^	1.77×10^-5^	7.95×10^-10^
F67	1.60×10^5^	2.33×10^-5^	1.45×10^-9^
HN3	4.03×10^5^	7.85×10^-5^	1.95×10^-9^

**Table 2:** IC_50_ (ng/ml), K_d_, and expression yield of GPC3 immunotoxins

	IC_50_ Hep3BLG	IC_50_ HepG2LG	IC_50_ Huh-7LG	IC_50_ G1LG	K_d_ (M)	*IC_50_(HCC) / log[K_d_ ×10^14^]	Yield in E.coli (mg/L)
J80A	7.9	3.1	2.5	1.2	3.50×10^-11^	1.2	0.3
C46	14.5	5.9	1.8	6.1	1.96×10^-10^	1.7	0.2
A43	18.1	3.1	1.9	1.2	1.52×10^-12^	3.5	1.5
G15	18.3	12.9	2.9	2.4	2.25×10^-10^	2.6	1.2
I82	21.5	6.9	2.1	0.8	4.05×10^-10^	2.2	5.0
J80B	21.8	7.7	1.8	2.8	2.95×10^-11^	3.0	3.0
A5	25.5	6.4	1.5	2.1	1.82×10^-11^	3.4	1.1
A18	33.5	9.3	7.6	2.7	2.14×10^-14^	50.9	0.6
J58	61.1	12.6	5.8	2.0	1.05×10^-10^	6.5	2.5
F5	93.6	31.5	24.3	0.9	2.28×10^-11^	14.8	1.4
H49	108.8	15.9	6.0	3.9	4.00×10^-10^	9.4	0.2
HN3	109.0	10.8	6.6	2.1	1.95×10^-9^	7.9	0.8
I34	117.9	43.3	34.5	1.6	5.04×10^-10^	13.8	4.0
I88	163.8	70.3	26.1	16.1	7.95×10^-10^	17.7	0.1
F67	164.4	39.6	18.5	2.1	1.45×10^-9^	14.3	0.1
R6	-	-	-	-	-	-	3.0

* IC_50_ (HCC) is the average IC_50_ values of HCC cell lines Hep3BLG, HepG2LG, and Huh-7LG.

## Abbreviations

ADC: antibody-drug conjugate
CAR-T: chimeric antigen receptor T cell
CTLA-4: cytotoxic T-lymphocyte-associated protein 4
FGFR4: fibroblast growth factor receptor 4
GPC3: Glypican-3
HCC: Hepatocellular carcinoma
HCL: hairy cell leukemia
PD-1: programmed cell death protein 1
SIRPα: signal-regulatory protein α
SPR: Surface plasmon resonance
VH: heavy chain variable region
